# Metabolic regulation of immune memory and function of microglia

**DOI:** 10.7554/eLife.107552

**Published:** 2025-10-10

**Authors:** Nikolaos Nirakis, Sofia Dimothyra, Eleftheria Karadima, Vasileia Ismini Alexaki

**Affiliations:** 1 Institute for Clinical Chemistry and Laboratory Medicine, Faculty of Medicine and University Hospital Carl Gustav Carus, Technische Universität Dresden Dresden Germany; Münster University Hospital Germany; National Institute of Immunology New Delhi India

**Keywords:** microglia, training, tolerance, cell metabolism

## Abstract

Innate immune cells possess memory-like properties. Exposure to infections or sterile inflammation can prime them, leading to either exacerbated inflammatory responses, a process called trained immunity, or reduced responsiveness to pro-inflammatory signals, a process termed immune tolerance. Microglia, the resident innate immune cells of the central nervous system, are central players in neurodegenerative diseases. Characterizing trained immunity and tolerance in microglia is necessary for a better understanding of neurodegenerative diseases. Cell metabolic processes orchestrate microglia inflammatory responses and promote epigenetic changes shaping immune memory in microglia. Here, we review current knowledge on the role of cell metabolic pathways in microglia innate immune memory formation, focusing on glucose, glutamine, and lipid metabolism. Moreover, we address the significance of microglial immune memory in disease pathology and discuss the potential of therapeutic targeting of cell metabolic pathways in neurodegenerative disorders.

## Introduction

Microglia, the resident immune cells of the central nervous system (CNS), derived from yolk sac progenitors during early embryogenesis, serve as vigilant sentinels, essential for maintaining CNS homeostasis (Eyo and Wu, 2019; Ginhoux et al., 2010). These unique cells continuously monitor the CNS, phagocytosing debris and modulating neuronal connections to support brain development (Kettenmann et al., 2011). Microglia are now recognized for their significant heterogeneity, rendering the M1/M2 polarization theory outdated (Masuda et al., 2020; Paolicelli et al., 2022). They acquire different transcriptional phenotypes: Hammond et al., 2019 identified 9 states, with microglia in young mice showing the greatest diversity, while Sun et al., 2023 identified 12, including homeostatic, inflammatory, and lipid-processing states in Alzheimer’s disease (AD). This dynamic diversity highlights the potential of microglia to adapt to specific CNS microenvironments in different (patho) physiological conditions (Dadwal and Heneka, 2024).

Microglia are long-lived cells; hence, they are exposed to multiple and sequential stimuli, which determine their phenotype and function. This process was initially termed microglia ‘priming’ and was especially associated with aging or disease (Neher and Cunningham, 2019; Dilger and Johnson, 2008). Later, the concept of innate immune memory emerged and reshaped our understanding of immune cell capabilities. Trained immunity, an adaptive-like memory in innate immune cells, results in a heightened response upon re-exposure to a pathogen or inflammatory stimuli (Netea et al., 2020). Conversely, immune tolerance dampens inflammatory responses upon re-exposure (Waldmann, 2016). These memory mechanisms are achieved through different signaling pathways, such as Toll-like receptor (TLR) signaling, cytokine-induced Janus kinase (JAK)/signal transducer and activator of transcription (STAT) signaling, β-glucan-induced Dectin 1 signaling, and AKT/mechanistic target of rapamycin (mTOR) signaling, as well as metabolic shifts and epigenetic reprogramming that establish long-term functional changes (Netea et al., 2020; Zhang et al., 2022). Metabolic pathways like glycolysis, oxidative phosphorylation (OXPHOS), and fatty acid oxidation (FAO) shape macrophage immune responses (O’Neill et al., 2016). Similarly, metabolic adaptations in microglia orchestrate their response to injury or infection and facilitate innate immune memory (Zhang et al., 2022; Wendeln et al., 2018; Bernier et al., 2020a). Upon activation, microglia undergo a metabolic shift toward glycolysis, which promotes pro-inflammatory responses (Baik et al., 2019; Suhail et al., 2023; Cheng et al., 2021; Lauterbach et al., 2019). In multiple sclerosis (MS), microglia adopt a phenotype with enhanced glycolysis, pro-inflammatory responses, and phagocytosis features during the early phase of demyelinated lesions, while during the later phase, they shift to enhanced OXPHOS (Qin et al., 2023). However, OXPHOS also increases acutely upon inflammatory activation of tissue macrophages and is required for the inflammatory response (Lauterbach et al., 2019; Wculek et al., 2023). Such metabolic adaptations not only regulate macrophage and microglial immediate responses but also foster ‘metabolic memory’ that shapes the response to future encounters (Bernier et al., 2020b). Long-term epigenetic modifications are key in the formation of innate immune memory (Ferreira et al., 2024). Metabolic intermediates are used as substrates for histone modifications determining gene expression implicated in innate immune memory (Lauterbach et al., 2019). For instance, acetyl-CoA, produced via glucose metabolism and fatty acid breakdown, is the most common substrate for histone acylations (Bhattacharya and Tu, 2024). Here, we review current knowledge on the regulatory networks of cell metabolism and epigenetics, which facilitate immune memory in microglia.

## Trained immunity

For decades, only adaptive immunity was considered to exhibit memory-like properties. Recently, innate immune cells were shown to acquire memory, a characteristic possibly evolved to protect organisms that lack adaptive immunity against reinfection (Bekkering et al., 2021). The concept that tissue-resident macrophages respond differentially after priming is known as peripheral trained immunity (Bekkering et al., 2021). The role of innate immune memory is cell- and context-dependent (Netea et al., 2020; Bekkering et al., 2016; El-Osta et al., 2008; Chakraborty et al., 2023; Wang et al., 2023; Hajishengallis et al., 2025). In cardiovascular disease, monocytes exhibit trained responses, driven by metabolic and epigenetic long-term modifications (Bekkering et al., 2016). Diabetes has also been linked with trained innate immune responses (El-Osta et al., 2008). In pathogen-induced lung inflammation, alveolar macrophages trained by previous bacterial exposure exhibit enhanced efferocytic capacity and promote inflammation resolution (Chakraborty et al., 2023). In sepsis, pro-inflammatory responses are amplified by trained immune cells (Wang et al., 2023). In cancer, trained immunity enhances the anti-tumor activity of innate immune cells (Hajishengallis et al., 2025). Microglia are the main drivers of innate immune memory in the brain across a spectrum of neurological diseases (Wendeln et al., 2018; Lan et al., 2024). Systemic administration of lipopolysaccharide (LPS) is widely used to prime microglia in mice (Wendeln et al., 2018; Alexaki, 2021; Alexaki et al., 2018). While LPS crosses to a minimal extent the blood–brain barrier (BBB), systemic LPS administration upregulates circulating cytokines such as TNF, IL-1β, and IL-6, which signal through the BBB, activate BBB-associated endothelial cells, or transpass the BBB to target microglia (Wendeln et al., 2018; Alexaki, 2021). Moreover, LPS (2–3 mg/kg) given systemically to mice can disrupt the BBB further facilitating the effects of peripheral inflammatory mediators (cytokines, lipids, etc.) on microglia (Lawrence et al., 2024; Banks et al., 2015). Hence, the LPS-induced immune memory in microglia is likely mediated by factors other than LPS per se.

**Figure 1.**
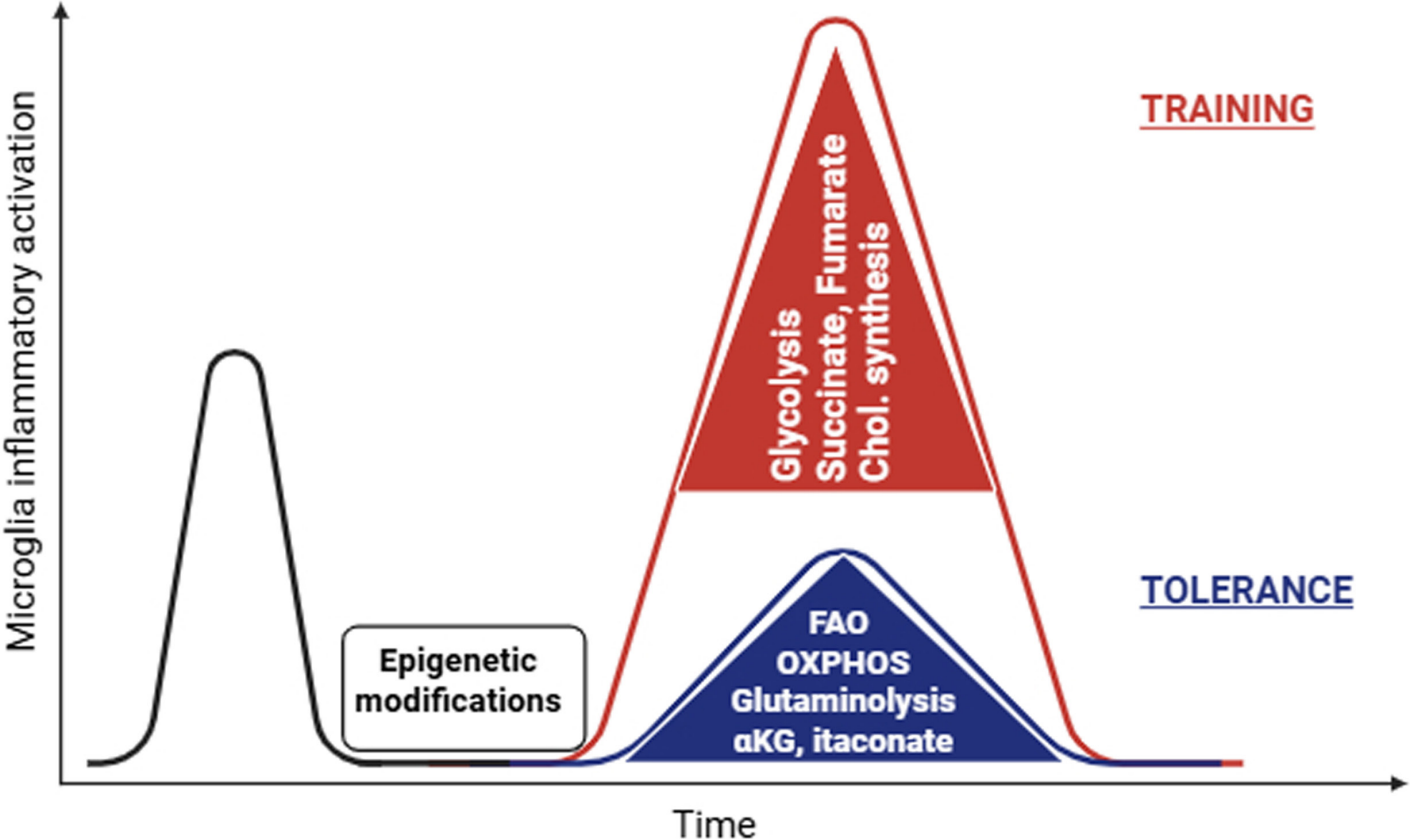
Cell metabolic reprogramming mediating microglia immune memory. Microglia can be trained (red) or tolerized (blue) exhibiting enhanced or dampened inflammatory responses, respectively (Wendeln et al., 2018; Feng et al., 2020; De Sousa et al., 2021; Dong et al., 2024). Epigenetic modifications, like H3K4me1 and H3K27ac, driven by cell metabolic reprogramming, facilitate development of innate immune memory (Wendeln et al., 2018; Arts et al., 2016; Liu et al., 2017a; McManus et al., 2025; Domínguez-Andrés et al., 2019). Glycolysis is induced in trained microglia and promotes neuroinflammation (Leng et al., 2022; Lepiarz-Raba et al., 2023; Fairley et al., 2023; Wang et al., 2021; Cheng et al., 2014). TCA cycle reprogramming is key in immune training and tolerance. Succinate promotes inflammation by activating the HIF-1α–IL-1β pathway (Tannahill et al., 2013) and fumarate increases H3K4me3 marks driving trained immunity (Arts et al., 2016). On the other hand, α-ketoglutarate (αKG), produced via glutaminolysis, and itaconate promote immune tolerance (Liu et al., 2017a; McManus et al., 2025). Cholesterol synthesis is upregulated in trained macrophages and mediates trained immunity (Arts et al., 2016; Domínguez-Andrés et al., 2019; Chen et al., 2022; Bekkering et al., 2018). On the other hand, FAO and OXPHOS are generally associated with a less inflammatory and more phagocytic microglial phenotype (Leng et al., 2022; Lepiarz-Raba et al., 2023; Fairley et al., 2023). Created with BioRender.com.

In the APP23 mouse model of AD, a single LPS dose (0.5 mg/kg) applied intraperitoneally (i.p.) at the age of 3 months increases synapse phagocytosis and exacerbates cerebral Aβ deposition 3 months later (Wendeln et al., 2018; Meng et al., 2024). Also, immune priming of microglia with one LPS dose aggravates neuroinflammation and progression of brain ischemia induced 1 month later (Wendeln et al., 2018; Feng et al., 2020; Figure 1). Similarly, sepsis causes a long-lasting trained phenotype in microglia, leading to increased Aβ-induced neuroinflammation (De Sousa et al., 2021). However, in other studies using the 5xFAD mouse model, a single LPS i.p. injection (1 mg/kg) at 6 weeks of age reduced microglia-mediated neuroinflammation and AD-like pathology 4.5 months later (Yang et al., 2023). The contradictive results in APP23 and 5xFAD mice may suggest potential influence of the animal model and age of the mice on the outcome of LPS-mediated microglia training but may also be caused by different types of LPS, differences in the microbiome, or other facility-dependent factors (Wendeln et al., 2018; Yang et al., 2023). Trained microglia with exacerbated immune response are also present in brain cancer patients exposed to radiation treatment (Voshart et al., 2024). Moreover, peripheral trained immune responses are triggered by stroke, promoting systemic inflammation and causing chronic cardiac dysfunction (Simats et al., 2024).

## Immune tolerance

Repetitive pro-inflammatory stimulation dampens the innate immune response upon re-exposure, a phenomenon termed immune tolerance. This mechanism has probably evolved to prevent excessive and deleterious, for healthy tissue, inflammation. In brain ischemia and neurodegeneration, microglia may acquire a tolerized phenotype, alleviating neuroinflammation (Liu et al., 2017b; Ma et al., 2023). In AD, microglia exhibit a tolerogenic phenotype driven by metabolic reprogramming (Baik et al., 2019). While a trained microglia state exacerbates AD, a tolerized one induced by LPS administered i.p. on 4 consecutive days in low doses (0.5 mg/kg) ameliorates AD pathology 3 months later (Wendeln et al., 2018). While the first injection caused a modest inflammation in the brain, the second LPS dose increased brain pro-inflammatory cytokines, while circulating cytokines dropped. After the fourth injections, brain cytokine levels also dropped with only IL-10 levels remaining elevated, indicating immune tolerance (Wendeln et al., 2018). Microglia tolerized by the same treatment in mice also reduce neuronal damage and neuroinflammation after brain ischemia (Wendeln et al., 2018). Divergent effects of repeated systemic LPS application on dopaminergic neuron loss were reported. While repeated LPS administration (0.5 mg/kg, i.p. on 3 consecutive days) was reported to protect the dopaminergic system in a mouse model of Parkinson’s disease (Dong et al., 2024), other studies showed that repeated LPS application induced loss of dopaminergic neurons 2 weeks after the last LPS treatment (Bodea et al., 2014). In Huntington’s disease, microglia possibly fail to become tolerant, retaining a vicious cycle of neuroinflammation (Steinberg et al., 2023). On the other hand, tolerized microglia are also linked to an inactive and dysfunctional state, contributing to disease pathology in mouse models of AD and ischemic stroke (Baik et al., 2019; Ma et al., 2023).

## Epigenetic modifications in microglia

Epigenetic modifications regulate the inflammatory responses of microglia and enable them to exhibit memory-like properties (Zhang et al., 2022; Yeh and Ikezu, 2019). Microglia are long-lived cells, implying a particularly relevant role of epigenetically regulated immune memory. Acetylation is a common histone modification typically activating gene transcription. It marks H3K9, H3K14, H3K18, H3K23, H3K27, and other histone sites in gene promoters or enhancers. Histone non-acetyl acylations, such as succinylation, propionylation, and crotonylation, occur less frequently (Bhattacharya and Tu, 2024). Histone lysine methylation also modifies gene expression leading to gene activation (di- or trimethylation of H3K4) or repression (H3K9me and H3K27me3). H3K4me3 marks mainly promoters, H3K4me1 is unique to enhancers, while other methylation sites such as H3K36 and H3K79 occur primarily in gene bodies (Bhattacharya and Tu, 2024; Caldwell and Li, 2024; Jambhekar et al., 2019). Methylation of histone arginine residues also occurs, but their association with gene expression is little understood (Jambhekar et al., 2019).

In mice, single or repetitive peripheral LPS stimulation inducing microglia training or tolerance, respectively, is related to differential presence of H3K4me1 and H3K27ac marks in microglia that persist for at least 6 months (Wendeln et al., 2018). In addition, neuronal cell death epigenetically promotes microglial phagocytosis in a brain region-dependent manner, being higher in cerebellar than striatal or cortical microglia (Ayata et al., 2018). Microglial clearing capacity is regulated by polycomb repressive complex 2 (PRC2), which epigenetically restricts gene expression related to clearance (Ayata et al., 2018). Moreover, methyl-CpG-binding protein 2 (MECP2) deletion increases histone H4 acetylation at regulatory regions of glucocorticoid- and hypoxia-induced genes, leading to enhanced inflammatory activation followed by increased depletion of microglia (Cronk et al., 2015). Accelerated aging resulting from deficiency of the DNA-damage repair protein ERCC1 (excision repair cross-complementing rodent repair deficiency, complementation group 1) drives gene expression via enrichment of the permissive marks H3K27ac and H3K4me3 in microglia (Zhang et al., 2022). On the other hand, combined deletion of histone deacetylases 1 and 2 (HDAC1 and HDAC2) results in increased H3K9ac and H3K27ac marks in microglia, enhanced Aβ phagocytosis, and improved cognition in an AD mouse model (Datta et al., 2018). Epigenetic changes facilitating immune memory in macrophages and microglia are significantly driven by cell metabolic changes (Netea et al., 2020). In the next paragraphs, we review the role of glucose, glutamine, and lipid metabolism in the context of epigenetic reprogramming and immune memory in microglia in health and disease. We focus on these metabolic pathways, as these are the best described in the context of microglial immune function and memory (Figure 1).

## Glucose metabolism

**Figure 2.**
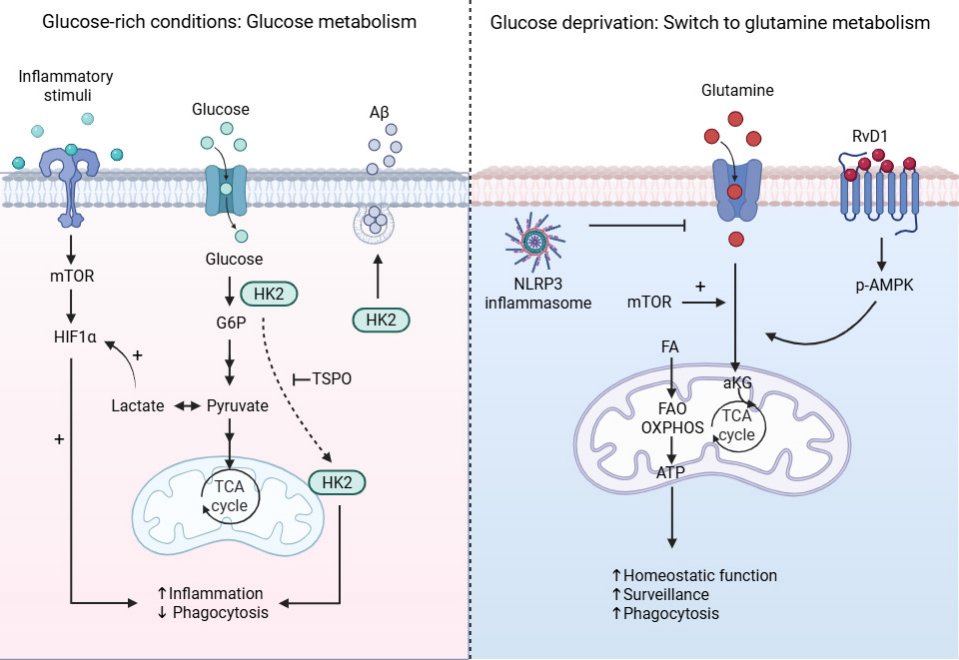
Glucose and glutamine metabolism in microglia. Under glucose-rich conditions (left), activated microglia exhibit enhanced glycolytic flux mediated by HK2 (Suhail et al., 2023; Fairley et al., 2023; Hu et al., 2022). Inflammatory activation triggers HK2 recruitment to mitochondria, a process regulated by TSPO, which further facilitates the glycolytic switch and inflammatory response of microglia (Fairley et al., 2023). In contrast, cytosolic HK2 promotes Aβ phagocytosis independent of its metabolic activity (Fairley et al., 2023). Lactate, the end product of glycolysis, stabilizes HIF-1α promoting a pro-inflammatory phenotype and attenuating phagocytosis (Dong et al., 2024; Zhang et al., 2023; Llibre et al., 2025; Magistretti and Allaman, 2018). Conversely, under glucose deprivation (right), microglia utilize glutamine as the main carbon source (Bernier et al., 2020a). Glutaminolysis fuels the TCA cycle with the generation of α-ketoglutarate (αKG), thereby boosting OXPHOS and enhancing ATP production (Bernier et al., 2020a; Bernier et al., 2020b). The oxidative metabolic profile is further supported by FAO and AMPK (Li et al., 2023a) and promotes a homeostatic microglial phenotype demonstrating enhanced surveillance and phagocytosis. Created with BioRender.com.

Upon inflammatory activation of macrophages and microglia, glycolysis is upregulated, a phenomenon similar to the Warburg effect in cancer cells (O’Neill et al., 2016; Cheng et al., 2021; Bernier et al., 2020b). This metabolic reprogramming is crucial for rapid energy production and biosynthesis of nucleotides, amino acids, and lipids, necessary for the immune response (O’Neill et al., 2016; Cheng et al., 2021; Yang et al., 2021). Increased glycolytic activity is a key feature of trained microglia (Wendeln et al., 2018; Figure 1). Interestingly, female mice with AD pathology show a more pronounced glycolytic shift than male AD mice, which is driven by 6-phosphofructo-2-kinase/fructose-2,6-biphosphatase 3 (PFKFB3) activation, leading to higher pro-inflammatory cytokine levels and decreased phagocytosis (Guillot-Sestier et al., 2021). Accordingly, inhibition of glycolysis and shifting to lipid oxidation and OXPHOS enhances phagocytosis in microglia (Leng et al., 2022; Lepiarz-Raba et al., 2023; Fairley et al., 2023). Hexokinase 2 (HK2), which catalyzes the first reaction of glycolysis, that is glucose phosphorylation to glucose 6-phosphate, is upregulated in neurodegenerative microglia (MGnD) (Hu et al., 2022; Krasemann et al., 2017). In 5xFAD mice, HK2 inhibition promotes Aβ phagocytosis, reduces amyloid deposition, and improves cognition (Leng et al., 2022). On the other hand, HK2 genetic deletion reduces energy production and suppresses microglial surveillance and damage-triggered migration, potentiating neuroinflammation and cerebral damage in ischemic stroke models (Hu et al., 2022). Mechanistically, it was shown that inflammatory activation or Aβ exposure triggers HK2 recruitment to mitochondria, which is required for the glycolytic switch and inflammatory response of primary microglia (Fairley et al., 2023). In contrast, cytosolic HK2 promotes phagocytosis independent of its metabolic activity (Fairley et al., 2023). Moreover, the end product of glycolysis, lactate, can act on microglia as a signaling molecule, stabilizing Hypoxia Inducible Factor 1 Subunit Alpha (HIF-1α), inducing histone modifications and altering gene expression (Dong et al., 2024; Zhang et al., 2023; Llibre et al., 2025; Magistretti and Allaman, 2018). The training-associated increase in glycolysis is mediated by the mTOR/HIF-1α pathway, while inhibition of AKT, mTOR, or HIF-1α blocks development of trained immunity in vitro in microglia and human monocytes (Wang et al., 2021; Cheng et al., 2014; Figure 2). In monocytes, β-glucan exposure induces a metabolic shift toward glycolysis and glutaminolysis, leading to elevated α-ketoglutarate and succinate (Arts et al., 2016). Succinate promotes inflammation in bone marrow-derived macrophages (BMDMs) by activating the HIF-1α–IL-1β pathway (Tannahill et al., 2013). Fumarate, the downstream metabolite of succinate, deactivates KDM5 histone demethylases, increasing H3K4me3 marks and driving trained immunity in monocytes in vitro (Arts et al., 2016). In contrast, α-ketoglutarate drives an anti-inflammatory phenotype by activating the H3K27 demethylase 6B (KDM6B, also known Jumonji domain-containing protein-3 (JMJD3)), thereby promoting endotoxin tolerance in BMDMs and potentially Aβ phagocytosis in primary microglia in vitro (Liu et al., 2017a; McManus et al., 2025; Figure 1).

Conversely, Aβ-induced tolerance in primary microglia involves sustained metabolic reprogramming toward OXPHOS with reduced glycolysis activity (Baik et al., 2019; Figure 1). This shift is mediated by HIF-1α and Myc downregulation and enhanced mitochondrial biogenesis mediated by PPARγ-peroxisome proliferator-activated receptor gamma coactivator 1 alpha (PGC-1α) (Baik et al., 2019; Jamwal et al., 2021). Negative feedback mechanisms regulate the inflammatory response and contribute to immune tolerance. For instance, pyruvate kinase M2 (PKM2) activation in inflammatory macrophages leads to glycolytic production of ATP, which is extracellularly converted to adenosine; the latter activates the adenosine receptor A2a and increases IL-10 and OXPHOS (Toller-Kawahisa et al., 2025). Moreover, itaconate produced in inflammatory macrophages and microglia via decarboxylation of cis-aconitate by the enzyme aconitate decarboxylase 1 (ACOD1) exerts anti-inflammatory effects through different pathways, including inhibition of succinate dehydrogenase (SDH), covalent modification of glycolytic enzymes and impairment of glycolysis, activation of NFE2 Like BZIP Transcription Factor 2 (NRF2), modulation of the NF-kappa-B inhibitor zeta (IκBζ)-Activating Transcription Factor 3 (ATF3) signaling and alkylation of gasdermin D leading to inflammasome inhibition (Li et al., 2023b; Liu et al., 2025; Bambouskova et al., 2021; Bambouskova et al., 2018; Lampropoulou et al., 2016; Mills et al., 2018; Qin et al., 2019). Itaconate confers endotoxin tolerance through SDH inhibition, while β-glucan promotes monocyte training via ACOD1 inhibition (Domínguez-Andrés et al., 2019; Figure 1). Moreover, itaconate binds to the same site on TET2 as the co-substrate α-ketoglutarate, inhibiting TET2 and decreasing the levels of 5-hydroxymethylcytosine, thereby suppressing expression of pro-inflammatory genes in macrophages and conferring tolerance against sepsis (Chen et al., 2022). In aged microglia, prostaglandin E2 (PGE2), a major mediator of inflammation, reduces glucose flux and mitochondrial respiration by promoting glycogen production via activation of the AKT–GSK3β–GYS1 pathway, thereby creating an energy-depleted, maladaptive pro-inflammatory state (Minhas et al., 2021). This energy-deficient state is further augmented by the enhanced dependence of aged myeloid cells on glucose as an energy source (Minhas et al., 2021). Concluding, trained microglia exhibiting increased inflammatory responses cover their energetic demands via increased glycolysis, while microglia tolerance is linked with enhanced phagocytosis and increased lipid oxidation and OXPHOS. Whether a shift in either direction proves beneficial or detrimental is highly context-dependent.

## Glutamine metabolism

Although microglia are highly glycolytic, glucose deprivation does not affect their surveilling function. This is due to their capacity to rapidly adapt to the absence of glucose by using glutamine as an alternative metabolic fuel (Bernier et al., 2020a). Glutaminolysis, along with FAO, sustains OXPHOS in microglia in aglycemia and supports the maintenance of microglial surveillance via mTOR signaling (Bernier et al., 2020a; Bernier et al., 2020b). Resolvin D1 (RvD1) enhances glutamine uptake and glutaminolysis in a 5′ AMP-activated protein kinase (AMPK)-dependent manner, supporting OXPHOS, increasing microglial phagocytosis of apoptotic neutrophils, and alleviating neuroinflammation after ischemia/reperfusion brain injury (Li et al., 2023a). Glutamine uptake in microglia is also regulated by amyloid deposition, which downregulates the expression of the glutamate–aspartate transporter SLC1A3 via inflammasome activation (McManus et al., 2025; Figure 2). α-Ketoglutarate produced via glutaminolysis is key for engagement of FAO and JMJD3-dependent epigenetic reprogramming driving anti-inflammatory responses and endotoxin tolerance in macrophages (Liu et al., 2017a). On the other hand, fumarate deriving from glutamine anaplerosis drives trained immunity in monocytes through epigenetic reprogramming (Arts et al., 2016). In sum, microglia demonstrate metabolic flexibility shifting in aglycemic conditions from glucose to glutamine metabolism to sustain beta oxidation and OXPHOS and thereby energetically support phagocytosis and eventually promote tolerance.

## Lipid metabolism

**Figure 3.**
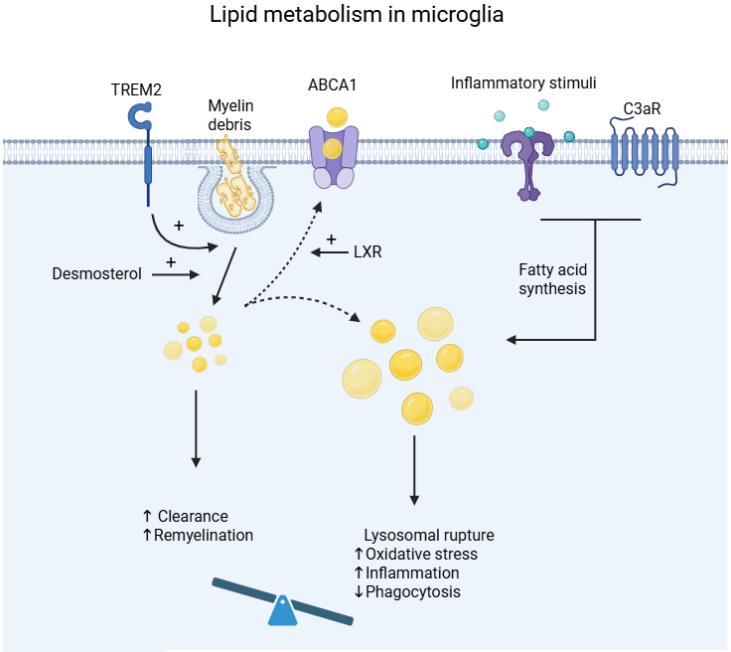
Lipid metabolism in microglia. Microglia process large amounts of lipids by phagocytosing myelin or cell debris (Poitelon et al., 2020). TREM2 is required for myelin clearance, cholesterol esterification, and lipid droplet formation (Gouna et al., 2021; Nugent et al., 2020). Desmosterol, the immediate precursor of cholesterol, activates LXR signaling, promoting myelin uptake, lipid efflux, and inflammation resolution (Berghoff et al., 2021). On the other hand, inflammatory signals such as C3aR activation can induce excessive lipid accumulation due to enhanced lipid synthesis and uptake causing lysosomal rupture, oxidative stress, inflammatory activation of microglia, and reduced phagocytosis (Marschallinger et al., 2020; Planas, 2024; Arbaizar-Rovirosa et al., 2023; Gedam et al., 2023; Loppi et al., 2023). Created with BioRender.com.

Microglia are challenged by a highly dynamic lipid-rich environment (Leyrolle et al., 2019; Penkert et al., 2021; Cantuti-Castelvetri et al., 2018). Through phagocytosis, they take up large amounts of lipids contained in cellular membranes and myelin (Poitelon et al., 2020). In disease, microglial lipid homeostasis adapts to their inflammatory response, phagocytosis, and regenerative function (Sun et al., 2023; Penkert et al., 2021; Shippy and Ulland, 2023; Litvinchuk et al., 2024). For instance, a subpopulation of lipid-processing microglia significantly increases in AD and correlates with the Αβ burden and cognitive decline, while cholesterol biosynthesis and metabolism is reprogrammed in tauopathy (Sun et al., 2023; Litvinchuk et al., 2024; Claes et al., 2021). Synthesis of desmosterol, the immediate precursor of cholesterol, in myelin-phagocytosing microglia in demyelinating lesions boosts Liver X receptor (LXR) signaling and myelin uptake and favors inflammation resolution and remyelination by oligodendrocytes (Berghoff et al., 2021; Figure 3). Mevalonate synthesis is upregulated in trained macrophages, and blocking mevalonate synthesis inhibits β-glucan-induced trained immunity (Arts et al., 2016; Bekkering et al., 2018). Mechanistically, mevalonate induces trained immunity in monocytes via Insulin-like Growth Factor 1 (IGF1)-R and mTOR activation, and histone modifications in pro-inflammatory genes (Bekkering et al., 2018). Accordingly, monocytes of hyper-immunoglobulin D syndrome patients exhibit heightened immune responses due to mevalonate kinase deficiency and mevalonate accumulation (Bekkering et al., 2018). Also in hematopoietic progenitors, β-glucan-induced training activates the cholesterol biosynthesis pathway by upregulating the expression of the rate-limiting enzyme HMG-CoA reductase (*Hmgcr*) (Mitroulis et al., 2018).

Lipid droplets accumulate in inflammatory microglia due to heightened lipid synthesis and increased lipid uptake (Marschallinger et al., 2020; Planas, 2024; Arbaizar-Rovirosa et al., 2023). Lipid droplet accumulation is mediated by complement 3a receptor (C3aR), which impairs Αβ phagocytosis and cognition in the APP-KI mouse model (Gedam et al., 2023; Figure 3). Inhibition of lipid synthesis ameliorates pathology in an AD mouse model, as toxic lipid secretion induced by activation of the integrated stress response mediates AD neurotoxicity (Flury et al., 2025). In parallel, FAO is upregulated in inflammatory activated microglia, as shown in the middle cerebral artery occlusion mouse model (Loppi et al., 2023). FAO-generated acetyl-CoA serves as a substrate for histone acetylation, which can mediate trained immunity, as shown in macrophages (Bekkering et al., 2018).

Triggering receptor expressed on myeloid cells 2 (TREM2) is a crucial lipid sensor (Wang et al., 2015). TREM2-deficient microglia fail to cluster around β-amyloid plaques, which leads to augmented amyloid deposition and neuronal loss (Wang et al., 2015). TREM2 is also required for cholesterol esterification facilitating myelin clearance by microglia and thereby remyelination (Gouna et al., 2021). Moreover, *Trem2^−/−^* microglia fail to clear myelin cholesterol due to defects in cholesterol efflux and accumulate cholesteryl esters (CE) in the form of lipid droplets (Nugent et al., 2020; Figure 3). In addition, TREM2 mediates LPS-induced microglia tolerance related to synapse phagocytosis but not inflammation (Meng et al., 2024) and supports the survival of microglia associated with amyloid plaques by promoting glucose uptake in microglia (Götzl et al., 2019; Xiang et al., 2021). Similarly, CD300f, a TREM2-like lipid-sensing immune receptor, preserves glucose uptake and microglial metabolic fitness in mice, while its deficiency leads to frailty, associated with cognitive decline and depressive-like behaviors, particularly in female mice (Evans et al., 2023; Lago et al., 2020). On the other hand, TREM2 induces Apolipoprotein E (ApoE) signaling and plays a key role in the passage of homeostatic microglia to MGnD in mouse models of AD, MS, and amyotrophic lateral sclerosis (Krasemann et al., 2017). The ApoE4 allele is the strongest genetic risk factor for late-onset AD and ApoE-driven acquisition of the MGnD phenotype associates with loss of the tolerogenic function of microglia in neurodegenerative disease (Krasemann et al., 2017; Lee et al., 2023). ApoE4 promotes CE accumulation in microglia, while increasing lipid efflux by LXR agonism or ATP-binding cassette transporter A1 (ABCA1) overexpression ameliorates tau pathology and neurodegeneration in P301S/ApoE4 mice (Litvinchuk et al., 2024). However, excessive uptake of cholesterol-rich myelin debris overwhelms the efflux capacity of aged microglia, resulting in cholesterol crystal formation and phagolysosomal rupture stimulating inflammasome activation, while stimulation of reverse cholesterol transport restores the remyelinating capacity of aged mice (Cantuti-Castelvetri et al., 2018; Figure 3).

Microglial maturation and function are also influenced by gut microbiota via short-chain fatty acids (SCFAs) (Colombo et al., 2021; Erny et al., 2021). Germ-free (GF) mice display an immature microglia phenotype, epigenetically determined by H3K4me3 and H3K9ac modifications, which is associated with respiratory chain defects due to impaired complex II activity (Erny et al., 2021). Acetate, a microbiome-derived SCFA, restores mitochondrial function in microglia of GF mice, exerts anti-inflammatory effects, and regulates microglial phagocytosis potentially by modulating histone deacetylase activity (Erny et al., 2021; Caetano-Silva et al., 2023). However, GF mouse models of AD present milder disease progression, with reduced cognitive deficits due to enhanced microglial Aβ clearance (Colombo et al., 2021; Harach et al., 2017; Mezö et al., 2020). SCFA supplementation reverses the protective phenotype of GF mice by increasing the Aβ load and impairing amyloid clearance (Colombo et al., 2021).

In summary, lipid metabolism plays a central role in microglia function and memory formation. Balance between lipid accumulation and cholesterol efflux allows for proper clearance, while excessive lipid accumulation leads to cell damage, inflammation, reduced phagocytosis, and overall perturbed function.

## Conclusions

Concluding, glycolysis, OXPHOS, glutaminolysis, TCA cycle reprogramming, FAO, cholesterol metabolism, and peripheral SCFA form metabolic networks that determine epigenetic modifications driving immune memory formation in microglia. Identifying key regulatory nodes in these networks could pave the way for novel therapeutic strategies in neurodegenerative diseases. Modulation of the glycolytic flux, OXPHOS, glutaminolysis, and cholesterol metabolism reprogrammed microglia function and ameliorated disease in preclinical studies (Baik et al., 2019; Leng et al., 2022; Lepiarz-Raba et al., 2023; Fairley et al., 2023; Hu et al., 2022; Litvinchuk et al., 2024; Berghoff et al., 2021). Moreover, repurposing metabolic drugs may offer additional possibilities. Metformin, an anti-diabetic drug, demonstrated neuroprotective effects in chronic experimental autoimmune encephalomyelitis by modulating microglial activation through selective inhibition of complex I and impeding reverse electron transport and ROS production (Peruzzotti-Jametti et al., 2024). Microglia immune training exacerbating brain damage in stroke can be reversed by mesenchymal stem cell therapy through downregulation of H3K4 methylation (Feng et al., 2020). Finally, leveraging sex-dimorphic metabolic differences in microglia might enable more precise, sex-specific therapeutic interventions (Guillot-Sestier et al., 2021).

## Limitations

While microglial immunometabolism and innate immune memory offer valuable insights, several limitations exist. Microglial heterogeneity across brain regions and disease states, as well as their dynamic nature, challenges the extrapolation of findings, as these cells display a spectrum of activation states rather than distinct phenotypes. Additionally, most studies primarily use rodent models, which may not fully represent human microglial function (Sabogal-Guáqueta et al., 2023). Moreover, the field lacks standardized protocols for inducing and measuring innate immune memory, leading to variable experimental outcomes, while existing models may not adequately mimic ‘real-life’ microglial training or tolerance in health and disease. Addressing these limitations is crucial for advancing therapeutic strategies for neurodegenerative diseases. Finally, we describe here disease-associated metabolic adaptations of microglia that may not directly mediate mechanisms of immune memory. However, as these metabolic changes determine microglia function over long time periods, they should be considered in the context of microglia immune memory.
